# Comparison of malaria incidence rates and socioeconomic-environmental factors between the states of Acre and Rondônia: a spatio-temporal modelling study

**DOI:** 10.1186/s12936-019-2938-0

**Published:** 2019-09-04

**Authors:** Meyrecler Aglair de Oliveira Padilha, Janille de Oliveira Melo, Guilherme Romano, Marcos Vinicius Malveira de Lima, Wladimir J. Alonso, Maria Anice Mureb Sallum, Gabriel Zorello Laporta

**Affiliations:** 1 Setor de Pós-graduação, Pesquisa e Inovação, Centro Universitário Saúde ABC, Fundação do ABC, Santo André, SP Brazil; 2 Gerência Estadual de Controle de Endemias, Rio Branco, AC Brazil; 3 Departamento de Epidemiologia, Faculdade de Saúde Pública, Universidade de São Paulo, São Paulo, SP Brazil; 4 School of Forest Resources and Conservation, University of Florida, Gainesville, FL USA; 5 Cartagena, Spain

**Keywords:** Tropical forest, Deforestation, Spatio-temporal models, Dynamics models, Malaria distribution

## Abstract

**Background:**

*Plasmodium falciparum* malaria is a threat to public health, but *Plasmodium vivax* malaria is most prevalent in Latin America, where the incidence rate has been increasing since 2016, particularly in Venezuela and Brazil. The Brazilian Amazon reported 193,000 cases in 2017, which were mostly confirmed as *P. vivax* (~ 90%). Herein, the relationships among malaria incidence rates and the proportion of accumulated deforestation were contrasted using data from the states of Acre and Rondônia in the south-western Brazilian Amazon. The main purpose is to test the hypothesis that the observed difference in incidence rates is associated with the proportion of accumulated deforestation.

**Methods:**

An ecological study using spatial and temporal models for mapping and modelling malaria risk was performed. The municipalities of Acre and Rondônia were the spatial units of analysis, whereas month and year were the temporal units. The number of reported malaria cases from 2009 until 2015 were used to calculate the incidence rate per 1000 people at risk. Accumulated deforestation was calculated using publicly available satellite images. Geographically weighted regression was applied to provide a local model of the spatial heterogeneity of incidence rates. Time-series dynamic regression was applied to test the correlation of incidence rates and accumulated deforestation, adjusted by climate and socioeconomic factors.

**Results:**

The malaria incidence rate declined in Rondônia but remained stable in Acre. There was a high and positive correlation between the decline in malaria and higher proportions of accumulated deforestation in Rondônia. Geographically weighted regression showed a complex relationship. As deforestation increased, malaria incidence also increased in Acre, while as deforestation increased, malaria incidence decreased in Rondônia. Time-series dynamic regression showed a positive association between malaria incidence and precipitation and accumulated deforestation, whereas the association was negative with the human development index in the westernmost areas of Acre.

**Conclusion:**

Landscape modification caused by accumulated deforestation is an important driver of malaria incidence in the Brazilian Amazon. However, this relationship is not linearly correlated because it depends on the overall proportion of the land covered by forest. For regions that are partially degraded, forest cover becomes a less representative component in the landscape, causing the abovementioned non-linear relationship. In such a scenario, accumulated deforestation can lead to a decline in malaria incidence.

## Background

Human malaria emerged from the tropical forest of Africa, propagated globally and became a tropical and subtropical disease in the second half of last century [1–3]. Six species of *Plasmodium* parasites can cause disease in humans: *Plasmodium falciparum*, *Plasmodium vivax*, *Plasmodium malariae*, *Plasmodium ovale curtisi*, *Plasmodium ovale wallikeri* and *Plasmodium knowlesi* [1, 4–7]. Recently, *Plasmodium simium* emerged as another potential species to infect humans [8]. The malaria transmission cycle includes *Plasmodium* spp., anopheline and human components [9]. In 2017, 91 countries reported a total of 219 million cases of malaria, with 435,000 deaths [10]. Worldwide, *P. falciparum* malaria is more prevalent than *P. vivax* malaria. *Plasmodium falciparum* malaria accounted for 99.7% of the cases in areas across sub-Saharan Africa [10]. In the Americas, *P. vivax* malaria occurs more frequently than *P. falciparum* malaria [11–13], with 723,000 (74%) infections reported in 2017 [10]. Whereas *P. falciparum* causes higher levels of morbidity and mortality than *P. vivax* [14, 15], the latter is gaining attention as a major hurdle in the era of malaria elimination [16, 17]. A reason for this can be that current malaria commodities, including the available anti-malarial drugs, are not very effective against *P. vivax*, leading to a high proportion of *P. vivax* asymptomatic reservoirs that can infect anopheline vectors [13, 16], further propagating the parasites in environments where competent mosquito vectors occur.

The global malaria incidence rate has declined from 76 to 59 cases per 1000 population at risk from 2010 to 2017 [10]. However, the rate of decrease has either slowed or reversed in some regions since 2015 [10]. In the Americas (in 2017), malaria incidence has been increasing since 2013, mainly because of the Bolivarian Republic of Venezuela, Brazil and Nicaragua [10, 13]. Between 2016 and 2017, malaria incidence increased approximately 100% in Nicaragua and Venezuela. In 2017, Venezuela accounted for 53% of reported cases, followed by Brazil (22%) [10]. Malaria distribution is spatially clustered, with hotspots of transmission in Choco (in Colombia), Loreto (in Peru) and Bolivar (in Venezuela) [10, 18]. In Brazil, approximately 45% of reported cases are from 15 municipalities in the states of Acre and Amazonas [18].

In Brazil, malaria decreased by 65% from 2010 (384,655) to 2016 (133,591). However, the disease increased by 63% between 2016 and 2017 (217,928) in comparison to 2015 [10]. Most malaria cases occur in the Amazon River Basin. In 2017, 193,000 cases occurred in the Amazonian Region (99.95%), which were mostly *P. vivax* malaria (174,000; ~ 90%). Consequently, the majority of the studies have focused on hotspots of malaria transmission in areas across the Brazilian Amazon (e.g., [19–29]). Malaria transmission has been associated with several scenarios: (1) legal and illegal mining with high human exposure to mosquito bites, human movement and extensive environmental changes [16]; (2) expansion of agricultural frontiers, leading to deforestation, land-use changes and human encroachment in forested areas [30]; (3) discontinuity of malaria control programmes in poorly accessed remote areas [21]; and (4) ecological factors, which can drastically increase vector abundance, such as fish ponds in rural areas and towns [16, 25, 31]. These aforementioned transmission settings can represent transmission hotspots, and they were employed to construct a flexible model for predicting malaria emergence in similar scenarios [28, 32].

Frontier malaria is a concept offering an explanation for the trajectory of malaria incidence with deforestation and was applied for predicting the emergence of malaria in the Brazilian Amazon region [28]. This concept model predicts high malaria transmission risk in the first years of a human settlement in the Amazon forest. The main mechanisms are (1) a high number of immunologically naïve immigrants intermixed with asymptomatic human reservoirs, (2) a high contact rate between the main malarial vector and human hosts, and (3) a precarious socio-environmental matrix [28]. After 10 years of colonization and development in the settlement, the frontier malaria concept predicts a steep malaria decline rate. The mechanisms for the decline are related to overall improvements in the settlement with economic gains from agriculture, ranching and urban development [28].

**Fig. 1:**
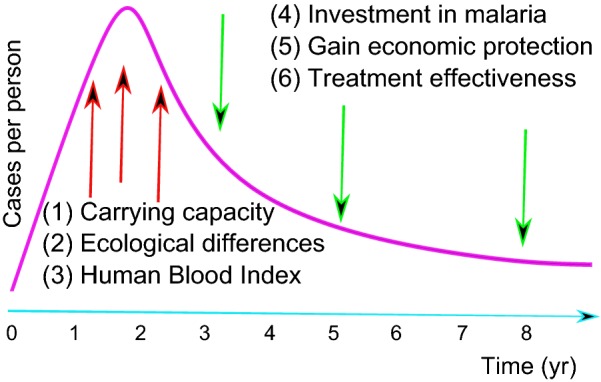
*Theoretical background*. The convex curve supports the convex trajectory observed in the generalization model [33] of the frontier malaria concept [28]. Environmental conditions (1–3) and socioeconomic factors (4–6) are processes estimated with parameters in the model by Baeza et al. [33]. Environmental conditions (1–3) are driving forces in the high-risk scenario of malaria transmission in the first years of colonization. Socioeconomic factors (4–6) counterbalance and surpass environmental conditions effects, decreasing malaria incidence in the long-term. (1) Carrying capacity: the maximum abundance of adult mosquitoes per unit of land area. (2) Ecological differences: the magnitude of land-use changes. (3) Human Blood Index: the proportion of blood meals from humans by a mosquito. (4) Investment in malaria: the effect of investment in malaria medication. (5) Gain economic protection: the rate which people gain protection against malaria due to the overall economic improvements. (6) Treatment effectiveness: the cost-effectiveness of the treatment

A mathematical model was developed to address the possible dynamical trajectories of malaria with land-use change in frontier regions [33]. This work is a theoretical generalization of the frontier malaria concept, with a mathematical model coupling land-use change, malaria transmission and economic development. Most of the plausible parameter space led to numerical simulations with malaria population dynamics showing an initial increase in malaria incidence followed by a decrease in this incidence afterwards [33]. The initial state of high malaria risk in early stages of land-use change is driven by environmental conditions. Malaria risk decreases over time because these environmental conditions interact with the socioeconomic factors that tend to reduce risk on slower and longer timescales [33]. The tension between environmental and socioeconomic forces supports the pattern of the rise and fall of malaria population dynamics under land transformation (Fig. 1).

Fragmented landscapes with approximately 30–70% forest cover have the forest fringe effect, which maximizes the abundance of the main malarial vector (*Anopheles darlingi*) in the Amazon [24]. Malaria transmission can be sustained in any given landscape with the forest fringe effect [34]. Landscapes near natural conservation units (e.g., federal forests and indigenous reserves) are generally represented by settlements with intermediate forest cover and present a high risk of malaria incidence [35]. The variation in malaria incidence associated with changes in forest cover (100–0%) can be depicted by a convex curve [36].

Herein, a test of the unimodal (i.e., convex curve) relationship between malaria incidence and forest cover on a large scale (Amazonian states) is proposed. The importance of this work is the necessity of depicting the big picture and overall knowledge of malaria transmission for tailoring interventions. Determinants of the disease in two Amazonian states (Rondônia and Acre) that share a common historical root and started colonization at the same time in the 1900s were addressed. The dissimilarity between the two is that Rondônia represents a deforested Amazonian state, while Acre represents a forest-conserved Amazonian state. In addition, the state of Rondônia was the epicentre of the malaria burden in the 1980s to 1990s, but this state has recently seen a strong decrease in its incidence rate [23, 28]. In contrast, in the state of Acre, transmission is stable with some areas defined as hotspots of malaria in Brazil [25, 29]. The hypothesis is that this difference in the transmission level is related to the following: (1) most of the area of Rondônia previously covered by forest has been deforested [30]; and (2) the state of Acre, which has larger areas of preserved forest, is under anthropogenic changes in the natural environment, and forest fragmentation is increasing in some regions [30]. The specific aims are as follows: (1) to analyse the spatio-temporal distribution of the incidence rates and compare them between the states of Acre and Rondônia (in the western Brazilian Amazon); and (2) to address potential determinants of the disease.

## Methods

### Study area

**Fig. 2:**
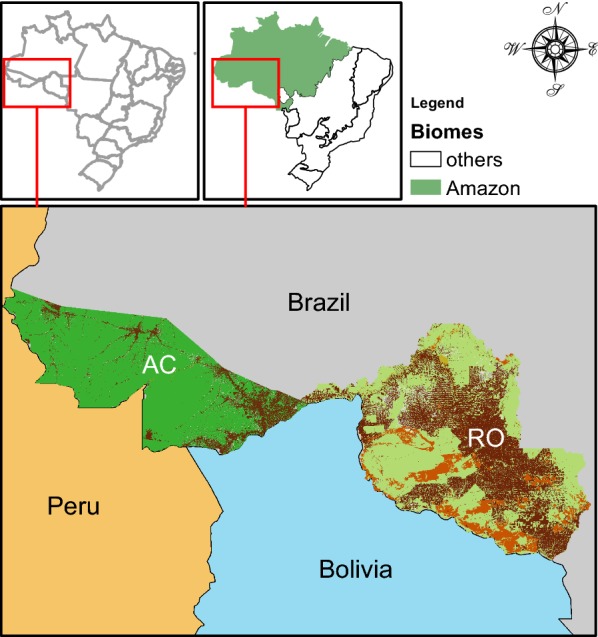
*Study region*. The Brazilian states of Acre (AC) and Rondônia (RO) are located in the Southwestern Amazon, bordering neighbouring Peru and Bolivia. Forest cover and fragmentation of these states are represented as dark/light green (forest), dark brown (deforested area) or light brown (rocky soil)

Acre and Rondônia states (Fig. 2) have a common historical root. The creation of both states is rooted, in part, in the Treaty of Petropolis signed in 1903 between Brazil and Bolivia. This agreement resulted in the end of a deadlock with respect to a Bolivian territory, which is now the geographical seat of Acre in Brazil, and allowed for the construction of the Madeira Mamoré Railroad, which gave rise to the city of Porto Velho, the capital of Rondônia.

These states have different assumptions for the colonization process. The pride of the people of Acre is latent in its history, which is the sum of the struggles of rubber workers, indigenous people, pioneers and descendants of individuals with these origins. Porto Velho, however, does not seem to neither feed from the cradle of its Amazonian history nor seek the past glory of the pioneers who had been there before.

The state of Rondônia (Fig. 2; 237.765 km^2^) shows a diversified phytogeography that reflects the heterogeneity of physical aspects such as relief, lithology, soil and climate. With the growing population, the tropical rain forest has been gradually decreasing since the late 1970s. Currently, natural forest is restricted to reserves, indigenous lands and parks. The mapping of the state of Acre (Fig. 2; 164.123 km^2^) shows the occurrence of highly preserved vegetation types with ombrophylous forest and campinarana (Amazonian plain forest). The climate in both states is humid tropical, with two major seasons: the rainy season from November to April and the dry season from May to October. Malaria incidence is higher in the rainy season because of the increase in available larval habitats for the mosquito vector.

The state of Rondônia has a mostly rural population (70%) out of a total of 1.7 million people estimated in 2018, who own approximately 905,000 vehicles (e.g., cars, trucks, buses and motorcycles). The average monthly income is US$251 per capita, and the human development index (HDI) is 0.69. In contrast, in Acre, the estimated population in 2018 was 800,000, with 70% living in rural areas. The number of vehicles in the whole state was 251,000, the income was lower (US$202 per capita), and the HDI was 0.66 (http://www.ibge.gov.br).

### Study design and rationale

This is an ecological study in epidemiology that employs aggregate malaria, environmental and socioeconomic data from January 2009 to December 2015 of all municipalities in the states of Acre and Rondônia, Amazon Region, Brazil. Malaria time-series data were first analysed, and monthly incidence rates were compared between Rondônia and Acre with EPIPOI v. 15 (Alonso and McCormick, Oxford, UK) [37]. The stationarity of the time-series malaria data was verified using an augmented Dickey-Fuller test with the package *tseries* in the R programming environment v. 3.5.1 (The R Foundation, Vienna, Austria) [38].

A second round of analysis was performed to correlate annual malaria incidence rates with annual accumulated deforestation from 2009 to 2015 for each state. To reduce the spatial dimension of 22 municipalities in Acre and 52 municipalities in Rondônia, the first axes of principal component analyses were utilized. These axes represent variations of malaria incidence and accumulated deforestation in each state. A Pearson’s product-moment correlation in R v.3.5.1 was applied to test the relationship between these variables.

A standard protocol of spatial analysis with geographically weighted regression (GWR) was employed for assessing the local correlation between annual malaria incidence rates and annual accumulated deforestation in each municipality of both states, using overall data from 2009 to 2015. A time-series modelling analysis was employed to verify the association between variations in monthly malaria incidence rates and climate, landscape, and social factors. This analysis was applied to those localities with the highest incidence rates in Acre.

### Malaria incidence rate

The malaria incidence rate was estimated as the number of malaria cases per 1000 population at risk. Data from each municipality in the states of Acre and Rondônia were downloaded from the SIVEP-Malaria database, available at http://portalms.saude.gov.br/saude-de-a-z/malaria/notificacao. The raw data were concatenated in a database for the analyses.

The estimated population of each municipality was available in the SIVEP-Malaria database. Because monthly based data were also needed, linear interpolation between subsequent years was performed using the following equation:

$$\frac{{y - y_{0} }}{{x - x_{0} }} = \frac{{y_{1} - y_{0} }}{{x_{1} - x_{0} }}$$

where *y*_*0*_ and *y*_*1*_ were the available population data in *x*_*0*_ and *x*_*1*_ months, respectively. The coordinate (*x*, *y*) was estimated, and the population data (*y*) were linearly interpolated in each month (*x*).

### Annual accumulated deforestation

To calculate the overall accumulated deforestation in km^2^ that occurred in a certain year per municipality in both Amazonian states, we employed publicly available information from the Instituto Nacional de Pesquisas Espaciais (INPE) (INPE/PRODES Project website, http://www.dpi.inpe.br/prodesdigital).

### Spatial regression analysis

As a first step, an ordinary least square model (a non-spatial model) was fitted in R v.3.5.1:

$$Y = \beta_{0} + \beta_{1} X + \varepsilon$$

where Y = annual malaria incidence rate (cases/pop*1000) and X = annual accumulated deforestation (%). Parameters $\beta_{0}$ = Y value when X equals zero, $\beta_{1}$ = linear effect of annual accumulated deforestation on annual malaria incidence rate, and $\varepsilon$ = model residuals. The statistical significance level was 5%.

To check whether the linear relationship between Y and X was not biased by the spatial dimension, residuals of the aforementioned linear model were tested for spatial autocorrelation with the Moran index calculation in GeoDa v. 1.12 (The University of Chicago, Chicago, Illinois, US)

$$I = \frac{n}{W}\frac{{\sum_{i} \sum_{j} w_{ij} z_{i} z_{j} }}{{\sum_{i} z_{i}^{2} }}$$

where *I* = Moran index (equivalent to the product $\frac{n}{W}$ $\frac{{\sum_{i} \sum_{j} w_{ij} z_{i} z_{j} }}{{\sum_{i} z_{i}^{2} }}$), *n* = number of municipalities, *W* = first-order Queen-type spatial weight matrix, $w_{ij}$ = element in spatial weights matrix, and $z_{i}$ and $z_{j}$ = deviations from the mean *z*. The statistical significance level was 5%.

When the non-spatial model was not adequate, the GWR was applied to model spatially heterogeneous relationships between Y and X in GWR v. 4.09 (Arizona State University, Tempe, Arizona, US).

$$Y\left( s \right) = \beta \left( s \right)X$$

where Y(s) = annual malaria incidence rate in each municipality and *β*(s)X = linear effect of annual accumulated deforestation on annual malaria incidence rate in each municipality.

### Time-series modelling

To verify the presence of stable foci of transmission in the state of Acre, a dynamic regression modelling analysis was performed. Socioeconomic, climate and landscape data were employed to verify the potential association of each factor to the incidence rate of malaria in the westernmost areas of Acre. The time-series of monthly malaria incidence data were modelled with the available socioeconomic-environmental data of the Cruzeiro do Sul (CZS), Mancio Lima (ML), Rodrigues Alves (RA), Porto Walter (PW) and Tarauaca (TA) municipalities from 2009 to 2015. These municipalities represent the current frontier malaria in the western Amazon.

Specifically, an autoregressive integrated moving average (ARIMA) model was utilized using the following equation:

$$y_{t} = \beta_{0} + \beta_{1} x_{1,t} + \cdots + \beta_{k} x_{k,t} + rY_{t - 1} + e_{t} + ae_{t - 1}$$

With the monthly malaria incidence rates as the response variables (*y*_*t*_), the socioeconomic-environmental factors (variables *x*_*1*_, *x*_*2*_,…, *x*_*k*_) were divided into three sets: (1) climate (2 variables); (2) landscape (2 variables); and (3) socioeconomic (5 variables). The implementation of ARIMA in the package *forecast* in R v. 3.5.1 [39] was utilized. Accordingly, the equation of the regression model was estimated using a stepwise approach with forward selection. The 95% confidence interval of each intercept (*β*_*1*_, …, *β*_*k*_) was estimated. The autoregressive parameter (*r*), the pure error (*e*) and the moving average (*a*) were also estimated. No assumptions on the lags for the socioeconomic-environmental factors were made. The covariate lags were selected based on the model’s best prediction. The ARIMA algorithm in the R *forecast* package automatically took seasonal differences (i.e., interannual variation) into account when they were relevant in improving model prediction. The time-series analysis protocol is available in Additional file 1.

Total precipitation (mm) and average maximum temperature (°C) were selected because of their well-known importance for standing water as habitats of the mosquito vector. Precipitation and temperature data are available in the Instituto Nacional de Meteorologia (INMET; http://www.inmet.gov.br). Total precipitation and average maximum temperature in the rainy (Nov.–Apr.) and dry (May–Oct.) seasons were interpolated using data from the following meteorological stations: Uruguaiana (− 29.75, − 57.08), Corumba (− 57.67, − 19.02), Ponta Pora (− 55.71, − 22.55), Eirunepe (− 69.86, − 6.66), Labrea (− 64.83, − 7.25), Benjamin Constant (− 70.03, − 4.38), Cruzeiro do Sul (− 72.66, − 7.6), and Rio Branco (− 70.76, − 8.16) and Tarauaca (− 67.8, − 9.96). More information on how temperature and precipitation were interpolated is provided in Additional file 2.

Two landscape parameters were chosen because they represented a proxy for the presence of mosquito vector larval habitats: (1) annual forest cover (km^2^) and (2) annual accumulated deforestation (km^2^) per municipal area. These land-use land-cover variables were obtained from the aforementioned INPE/PRODES Project website.

Annual socioeconomic data were obtained from the PNUD/Atlas Project website (https://popp.undp.org), including infant mortality rate (per 1000 live births), proportion of people living in extreme poverty (% of people living on less than US$1.90 per day), proportion of people living in poverty (% of people earning less than US$3.75 a day), a measure of inequality of income (GINI index, 0–1, the most inequality = 1) and municipal HDI (MHDI) (0, minimum; 1, maximum). These parameters were selected because they can represent risk factors for human exposure to mosquito vector bites and malaria.

### Ethical issues

Regarding the Brazilian Institutional Review Board for protection of human subjects, the present study does not require approval for access to data. Any patient information was not publicly available in the SIVEP-Malaria platform. In addition, malaria data are part of the public domain according to the Brazilian Law of Information Access (12.527/2011).

## Results

### Malaria incidence rate

**Fig. 3:**
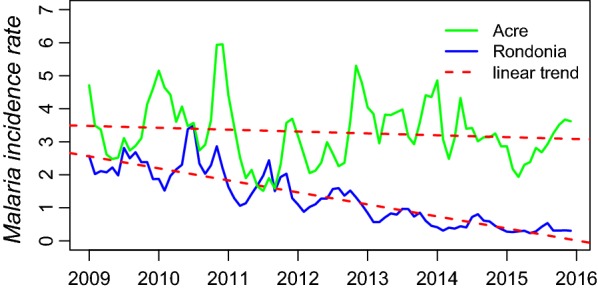
*Time series*. Malaria incidence rates in Acre and Rondônia

The malaria incidence rate ranged from 0.2 to 3.5 cases per 1000 population from 2009 to 2015, showing a decreasing linear trend (− 36%, P < 0.001) in Rondônia, whereas it ranged from 1.5 to 6 cases per 1000 population, without evidence of a linear trend (− 5%, P = 0.27) in Acre (Fig. 3). The results of the Dickey-Fuller test showed that the time-series of the malaria incidence rate in Rondônia had a stationary process (P < 0.01), whereas Acre had a non-stationary process (P = 0.11).

### Correlation

**Fig. 4:**
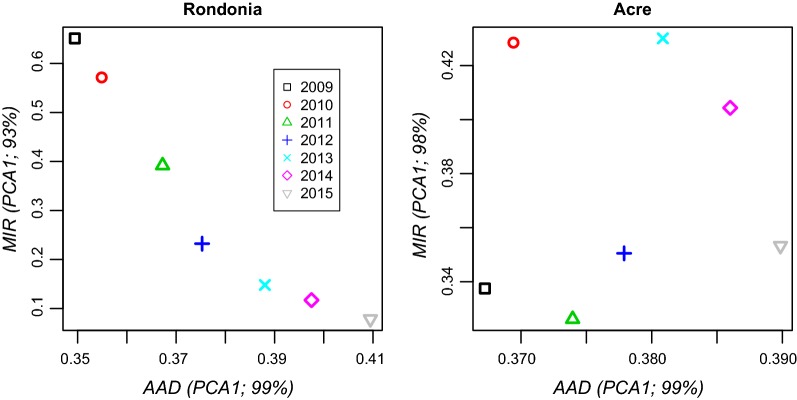
*Correlation testing*. Scatterplot of malaria incidence rate (MIR) vs. annual accumulated deforestation (AAD) in Rondônia and Acre. PCA1 = first axis of the principal component analysis that reduced all the municipality-based data into state-based data

The correlation between the malaria incidence rate and annual accumulated deforestation was strongly negative (i.e., more deforestation, less malaria) in Rondônia (r = − 0.96, P < 0.001), whereas it was not significant in Acre (r = 0.13, P = 0.79) (Fig. 4). In 2015, forest cover (85%) in Acre was 1.7 times higher than that estimated for Rondônia (51%), while the 2015 total deforested area (37%) in Rondônia was 2.84 times higher than that (13%) in Acre. The temporal processes of forest cover loss or gain per municipality in both states are depicted in Additional file 3. The full results of principal component analysis are in Additional file 4.

### Spatial regression analysis

**Fig. 5:**
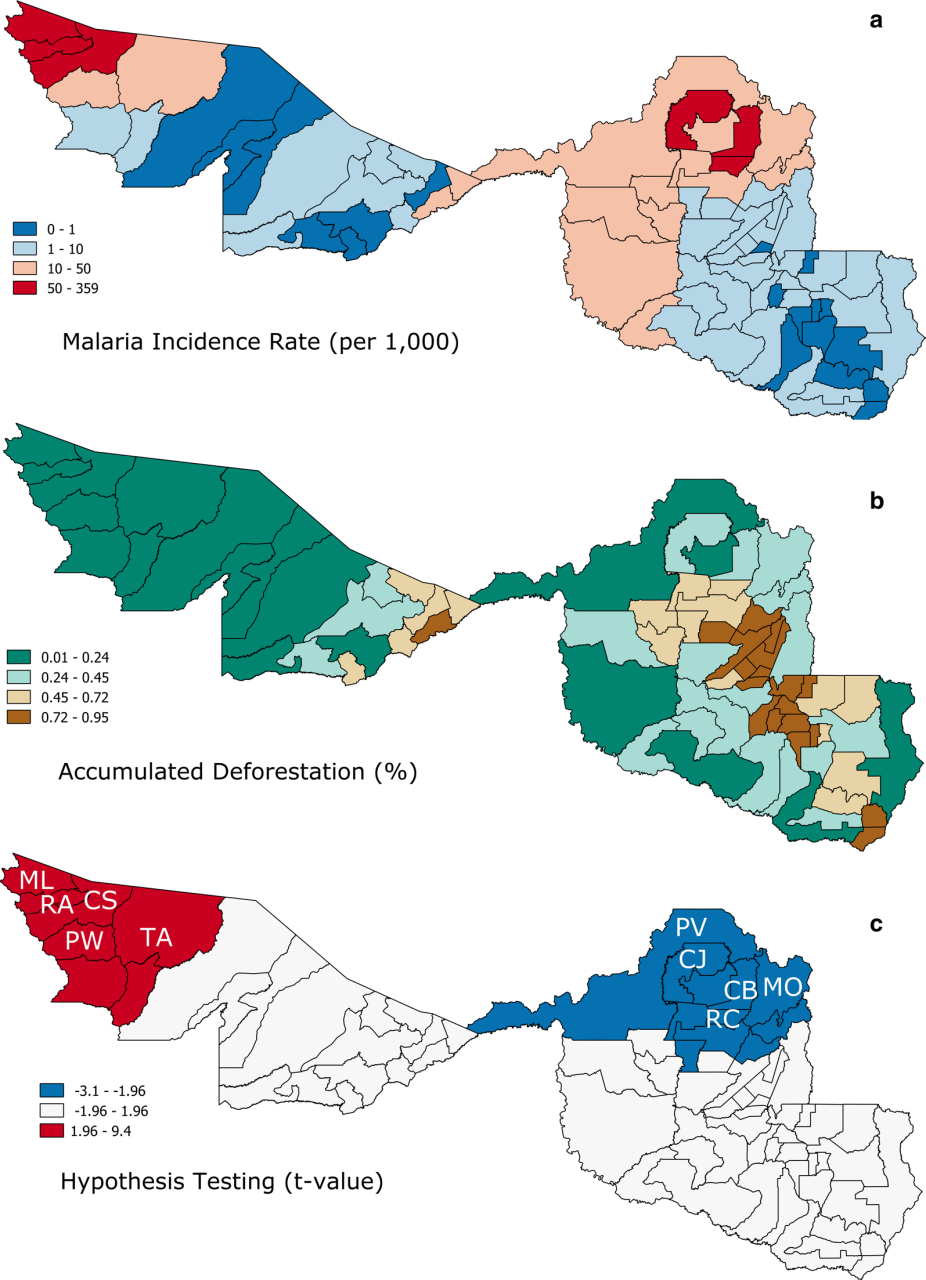
*Spatial analysis*. **a** Average malaria incidence rate 2009–2015 in each municipality (per 1000 inhabitants). **b** Accumulated deforestation in 2015 proportional to each municipality area. **c** Results of t-distribution from the geographically weighted regression model for each municipality. Acre municipalities: *ML* Mancio Lima, *RA* Rodrigues Alves, *CS* Cruzeiro do Sul, *PW* Porto Walter and *TA* Tarauaca; Rondônia municipalities, *PV* Porto Velho, *CJ* Candeias do Jamari, *CB* Cujubim, *RC* Rio Crespo, *MO* Machadinho d’Oeste

The non-spatial model comparing municipalities in Acre (22) and Rondônia (52) was not adequate because its residuals showed a strong spatial dependence (Moran’s I = 0.74, highly clustered). GWR showed that the relationship of malaria incidence (Fig. 5a) and annual accumulated deforestation (Fig. 5b) is complex because it could be either positive (i.e., more deforestation, more malaria; red cluster in Fig. 5c) or negative (i.e., more deforestation, less malaria; blue cluster in Fig. 5c), depending on the amount of remaining forest. Deforestation in areas with high forest cover, such as in Acre, showed a positive relationship with malaria incidence, whereas in areas with low forest cover (in Rondônia), additional deforestation decreased malaria incidence. The GWR model had better performance than the non-spatial model, with the coefficient of determination (*R*^2^) of 0.82 vs. 0.09 (non-spatial model) and Akaike information criteria (AIC) of 709 vs. 805 (non-spatial model).

### Time-series modelling

**Fig. 6:**
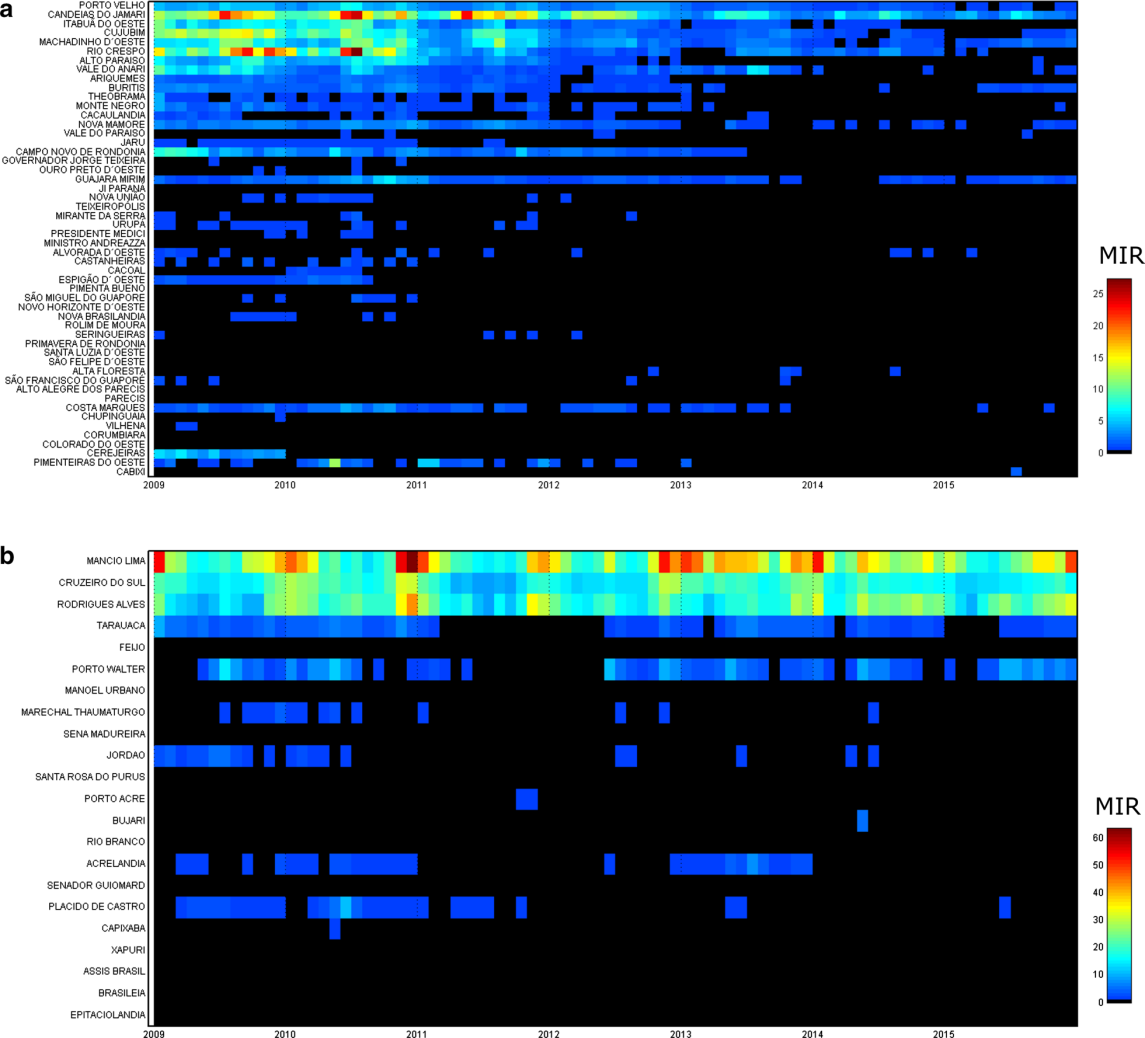
*Heat*-*grid time*-*series*. **a** Monthly malaria incidence rate per municipality 2009–2015 in Rondônia and **b** Acre. MIR = malaria incidence rate (cases/1000 people)

Malaria incidence rates decreased in the municipalities of Porto Velho, Candeias do Jamari, Itabua do Oeste, Cujubim, Machadinho d’Oeste and Rio Crespo in north-western Rondônia between 2009 and 2015 (Fig. 6a). However, in Mancio Lima, Cruzeiro do Sul, Rodrigues Alves, Porto Walter and Tarauaca, the monthly incidence ranged from 10 to 60 (per 1000 population) (Fig. 6b).

In the simple time-series regression analysis for the municipalities highlighted in Figs. 5c and 6 (ML, CZS, RA, PW, and TA), all socioeconomic-environmental factors were important predictors in the monthly variation in malaria incidence rates. Additionally, precipitation and temperature were seasonally correlated (i.e., more precipitation, lower temperature and vice versa), and accumulated deforestation and forest cover were positively correlated (which may reflect initial stages of colonization, as expected by the frontier malaria concept). All socioeconomic variables were correlated with each other but were only available in the Cruzeiro do Sul municipality. In the following analysis, precipitation and deforestation were selected to represent the environmental factors, while poverty and MHDI were selected to represent the socioeconomic factors. Complete results from the time-series modelling are in Additional file 5.

Multiple time-series regression analysis showed monthly malaria incidence rates as a function of precipitation, deforestation and MHDI or poverty in Cruzeiro do Sul (Table 1). In Cruzeiro do Sul, precipitation was positively but not statistically significantly correlated with malaria incidence, whereas deforestation and socioeconomic factors were statistically significant in the two models (Table 1). An increase of 0.01 in the MHDI meant 361 fewer malaria cases per 1000, whereas an increase of one unit in proportion (%) of people in poverty meant 346 more malaria cases per 1000. An increase in 10 km^2^ in deforestation meant ~ 400 more malaria cases per 1000.

**Table 1:** Results from the multiple time-series regression analysis of monthly malaria incidence rate, Cruzeiro do Sul-Acre, 2009–2015

	Estimate	SE	Pr(> |z|)
Model-1^a^			
Precipitation	0.24	0.16	0.14
Deforestation	42.24	18.91	0.026*
Poverty^1^	346.2	156.37	0.027*
Model-2^a^			
Precipitation	0.24	1.6	0.14
Deforestation	42.73	17.82	0.165
MHDI^2^	− 36,113	15,154	0.017

* Statistically significant result

^a^For the sake of simplicity time-series parameters’ estimations were omitted herein, but are available in the Additional file 5

^1^Proportion of people living in poverty

^2^MHDI: municipal human development index

In Mancio Lima, Rodrigues Alves, Tarauca and Porto Walter, deforestation is positively correlated with malaria incidence. These positive correlations are statistically significant in all cases, except in Tarauaca, where they are slightly non-significant (Table 2). An increase in 10 km^2^ in deforestation meant 2–54 more malaria cases per 1000.

**Table 2:** Results from the multiple time-series regression analysis of monthly malaria incidence rate, Mancio Lima, Rodrigues Alves, Tarauaca and Porto Walter, Acre, 2009–2015

	Estimate	SE	Pr(> |z|)
Mancio Lima^a^			
Precipitation	0.88	0.42	0.036*
Deforestation	5.44	1.32	< 0.001*
Rodrigues Alves^a^			
Precipitation	0.009	0.29	0.98
Deforestation	4.328	0.76	< 0.001*
Tarauaca^a^			
Precipitation	0.012	0.06	0.83
Deforestation	0.233	0.12	0.059
Porto Walter^a^			
Precipitation	− 0.001	0.004	0.97
Deforestation	1.32	0.24	< 0.001*

* Statistically significant result

^a^For the sake of simplicity time-series parameters’ estimations were omitted herein, but are available in the Additional file 5

## Discussion

The results of this study showed that the correlation between accumulated deforestation and malaria incidence can be discordant, showing either a positive or a negative statistical association. In Rondônia, the accumulated deforestation was three times higher than in Acre, and consequently, the trend in malaria incidence declined with increased deforestation. In contrast, the correlation was positive and statistically significant in Acre. Mechanistically, this pattern can be related to the frontier malaria concept [28] and the extension of this concept model by Baeza et al. [33], but it is also related to other works that state the importance of forest cover in malaria incidence in Amazon [24, 35, 36].

In the late 1970s, 2% of the state of Rondônia was deforested. Deforestation was intensified during the 1980s–1990s, affecting larger areas because of intensive migration. Malaria increased at very high rates during that time [16, 17]. However, starting in the late 1990s, Rondônia has gone through a turning point in its economic growth [40]. Mid-sized cities, which were merely a flow trail of natural resources to the urban centres of the capital (Porto Velho) or to southern Brazil in the 1980s, emerged as a central nerve in the production chain due to urban growth in the 2000s [40]. The five most important local hubs in Rondônia (Ji-Parana, Ariquemes, Vilhena, Cacoal, and Rolim de Moura) underwent population increases of 15–43% from 2000 to 2010 [40]. Capital investments that come to these urban centres in exchange for the region’s rich reserves of natural resources remain in the form of economic growth, rising socioeconomic indictors and public investments [40]. In addition, north-western Rondônia, which includes the capital (Porto Velho) and its adjacent municipalities (Fig. 5c), is considered a logging zone and a traditional wood transportation route in Brazil [30]. The fall of malaria observed in Rondônia can be related to both (1) socioeconomic factors that surpassed environmental forces on malaria transmission [28, 33] and (2) the loss of available habitats for the malarial vector due to deforestation [36].

Economic development in Acre is historically dependent on forest conservation for rubber exploitation and other extractivist activities, as well as fish farming [41]. Fish farming is not associated with deforestation [42] but can increase the risk of malaria [25, 31]. Cruzeiro do Sul was a former rubber town on the Jurua River and is now a local hub of economic growth and public investment in the westernmost area of Acre [40]. Additionally, Cruzeiro do Sul is also considered a local hub for the new frontier of logging zones [30]. The rise of malaria in the Jurua Valley Region may be related to environmental factors that tend to increase malaria risk in the early stages of colonization and to the lack of or still-incipient socioeconomic forces that tend to reduce malaria risk in the long term [33].

Parallel with the use of the frontier malaria concept [28] to predict malaria emergence in the Amazon is the debate regarding the association between deforestation in newly colonized sites and malaria emergence [43, 44]. The generality of the relationship between deforestation and malaria emergence was challenged [35] because the authors found higher malaria incidence in human settlements near priority areas for nature conservation. The controversy between the deforestation-malaria hypothesis [35] stimulated intensive debates [45, 46]. An alternative was proposed: deforestation may benefit or be harmful to the malarial vector population, depending on the pattern and proportion of forest cover [24].

**Fig. 7:**
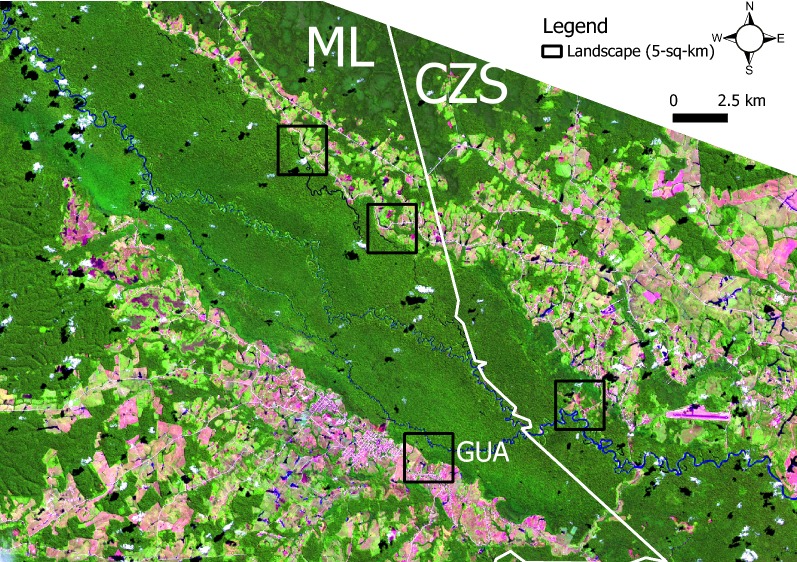
*Satellite imagery composite*. Landscape (5-km^2^) in where malaria transmission level [48] and the deforestation timeline [34] were estimated. *CZS* Cruzeiro do Sul, *ML* Mancio Lima, *GUA* Guarani-landscape studied by Lana et al. [49]. The satellite imagery composite was made by using the protocol developed by Ilacqua et al. [34] with QGis v. 2.18.14 (QGis Community, https://qgis.org) and SCP plugin v. 5.4.2 (Luca Congedo, Italy). Legend: blue, ground waters; dark green, forest vegetation; light green, crops, shrubs or secondary vegetation; pink, exposed or urban soil. Source: USGS/Landsat 8

The proposed unimodal relationship between forest cover and malaria emergence indicates that 30% to 70% of the remaining forest cover represents a landscape scenario that can encompass the ecological and environmental conditions that can favour peak transmission of malaria [24, 36, 47]. This risky scenario can occur either in newly colonized or old settlements [34]. For instance, the landscapes shown in Fig. 7 started colonization in the 1970s [34] and currently have high levels of transmission, with an estimated malaria incidence of 45–100 cases per day and a *P. vivax* reproduction number of 3.3–16.8 [48].

The satellite imagery composite shows Cruzeiro do Sul and Mancio Lima divided by a natural barrier: the hydrographic basin of the Moa River (Fig. 7). The configuration of the land use land cover shown in Fig. 7 can support an increase in malaria incidence [35] because of the availability of larval habitats for the malarial vector [24]. In addition, Lana et al. [49] identified improvements in socioeconomic factors in the landscape GUA (Fig. 7) at the same time as a high risk of malaria transmission due to (1) the abundance of malarial vectors and (2) the mobility of people in this urban centre of Mancio Lima. The pattern depicted in Fig. 7 seems supported by the frontier malaria concept [28] and Baeza et al. [33], thus representing the increasing phase of malaria population dynamics. Malaria decline may occur later in this real scenario (Fig. 7), when socioeconomic development can reduce transmission risk and accumulated deforestation can decrease larval habitat availability for the mosquito vectors.

The main malarial vector in the Amazon is *Nyssorhynchus darlingi*, formerly known as *Anopheles darlingi* [30, 48]. Foster et al. [50] built a globally based phylogeny of Anophelinae and concluded that Neotropical subgenera (including *Nyssorhynchus*) can be elevated to the genus level. In frontier malaria, *Ny. darlingi* is abundant, and its contact rate with humans is high [48]. On the one hand, other anopheline species known to be malarial vectors are not well adapted as *Ny. darlingi* in the anthropogenic matrix [51]. On the other hand, anopheline diversity continues to be underestimated in frontier malaria, with several species thought to be unknown [52]. Additionally, in specific scenarios, other species (e.g., *Nyssorhynchus albitarsis* sensu lato) can emerge as the primary vectors [53, 54].

A proposition for future research is herein made. The best study design for testing a temporal phenomenon as frontier malaria is a long-term prospective study. In the 1970s, a long-term prospective study was conceived for testing ecological theories (e.g., island biogeography) in the Amazon: the Forest Fragments Project (http://pdbff.inpa.gov.br/), e.g., [55]. Considering malaria elimination as a global target [56], the timing might be optimal for a bold proposal, such as a long-term prospective study on land transformation and its impact on socioeconomic and environmental determinants of malaria transmission.

## Limitation

Spatial and temporal variations in malaria incidence were not assessed by a statistical autoregressive model that considers time and space [57].

## Conclusions

Landscape modification caused by accumulated deforestation is an important driver of malaria population dynamics in Amazonia. In the initial phase of human settlement development, accumulated deforestation transforms a landscape with high forest cover into a landscape with intermediate levels of forest cover, increasing the odds of malaria emergence. In a later phase of development, when forest cover is reduced to low levels and its capacity to sustain malarial vectors’ larval habitats is decreased, the on-going accumulated deforestation only decreases the risk of malaria transmission.

The westernmost area of the state of Acre currently has stable malaria foci because it represents an initial phase of development, whereas the north-western area of the state of Rondônia, which had been considered the main hub for malaria in the 1980s and 1990s, is now seeing its malaria burden decline, which thus represents the later phase of development.

## Supplementary information

**Additional file 1.** Time-series analysis protocol in the R programming environment.

**Additional file 2.** Interpolation of total precipitation and average maximum temperature.

**Additional file 3.** Forest cover variations in municipalities of the states of Acre and Rondônia.

**Additional file 4.** Results from the principal component analysis.

**Additional file 5.** Results from the time-series modelling.

## Data Availability

The datasets used and analysed are of public domain, as detailed in the Methods section. They are available in the Additional files 1–5.

## Abbreviations

AAD: annual accumulated deforestation
AC: Acre state
AIC: Akaike information criteria
ARIMA: autoregressive integrated moving average
CZS: Cruzeiro do Sul municipality
GINI: gini index
GWR: geographically weighted regression
HDI: human development index
INMET: Instituto Nacional de Meteorologia
INPE: Instituto Nacional de Pesquisas Espaciais
MHDI: municipal human development index
MIR: malaria incidence rate
ML: Mancio Lima municipality
PNUD/UNDP: United Nations Development Programme
PRODES: Projeto de Monitoramento do Desmatamento na Amazônia
PW: Porto Walter municipality
RO: Rondônia state
RA: Rodrigues Alves municipality
SIVEP: Sistema de Informações de Vigilância Epidemiológica
TA: Tarauaca municipality
