# Supplementary material for: Comparison of malaria incidence rates and socioeconomic-environmental factors between the states of Acre and Rondônia: a spatio-temporal modelling study
Source: Malar J. 2019 Sep 4;18:306. doi: 10.1186/s12936-019-2938-0 (PMC6727495; doi:10.1186/s12936-019-2938-0)
Supplement: Supplementary file 1 — Additional file 1. Time-series analysis protocol in the R programming environment. [file 12936_2019_2938_MOESM1_ESM.docx]

Time-series analysis protocol in the R programming environment

The earlier version of this protocol was made by MVML. Now, GZL is the responsible for the last version.

We used R version 3.5.1 (2018-07-02). The following packages were installed and loaded:

#Install packages

install.packages("readxl"); install.packages('tseries'); install.packages("forecast");install.packages("lmtest")

#Load packages

require(readxl); require(tseries); require(forecast); require(lmtest)

We imported and visualized the database with malaria cases, population, environmental and social variables:

#Import data

CZS2 <- read_excel("CZS2.xlsx")

#Visualize data

View(CZS2)

We transformed the CZS2 data frame into a temporal class R object:

#Transformation to a temporal class data

czs.ts <- ts(CZS2, frequency = 12, start = c(2009,1), end = c(2015,12))

We performed descriptive analysis with summary and plot R-functions:

#Summary of data

summary(czs.ts); apply(czs.ts,2,sd)

#Plot temporal data

par(mfrow=c(3,4))

plot(czs.ts[,'cases']); plot(czs.ts[,'pop']); plot(czs.ts[,'coefcases']); plot(czs.ts[,'precipta']); plot(czs.ts[,'temp']); plot(czs.ts[,'forestcover']); plot(czs.ts[,'deforest']); plot(czs.ts[,'IMR']); plot(czs.ts[,'ExtP']); plot(czs.ts[,'Ppov']); plot(czs.ts[,'gini']); plot(czs.ts[,'MHDI'])

We tested the response variable for non-stationarity:

#Testing for non-stationarity

adf.test(czs.ts[,'coefcases'])

We performed simple dynamic regression models:

#Simple regression analysis

fit1<- auto.arima(czs.ts[,"coefcases"], xreg = czs.ts[,"precipta"], stepwise=FALSE, approximation=FALSE)

summary(fit1); coeftest(fit1)

fit2<- auto.arima(czs.ts[,"coefcases"], xreg = czs.ts[,"temp"], stepwise=FALSE, approximation=FALSE)

summary(fit2); coeftest(fit2)

fit3<- auto.arima(czs.ts[,"coefcases"], xreg = czs.ts[,"forestcover"], stepwise=FALSE, approximation=FALSE)

summary(fit3); coeftest(fit3)

fit4<- auto.arima(czs.ts[,"coefcases"], xreg = czs.ts[,"deforest"], stepwise=FALSE, approximation=FALSE)

summary(fit4); coeftest(fit4)

fit5<- auto.arima(czs.ts[,"coefcases"], xreg = czs.ts[,"IMR"], stepwise=FALSE, approximation=FALSE)

summary(fit5); coeftest(fit5)

fit6<- auto.arima(czs.ts[,"coefcases"], xreg = czs.ts[,"ExtP"], stepwise=FALSE, approximation=FALSE)

summary(fit6); coeftest(fit6)

fit7<- auto.arima(czs.ts[,"coefcases"], xreg = czs.ts[,"Ppov"], stepwise=FALSE, approximation=FALSE)

summary(fit7); coeftest(fit7)

fit8<- auto.arima(czs.ts[,"coefcases"], xreg = czs.ts[,"gini"], stepwise=FALSE, approximation=FALSE)

summary(fit8); coeftest(fit8)

fit9<- auto.arima(czs.ts[,"coefcases"], xreg = czs.ts[,"MHDI"], stepwise=FALSE, approximation=FALSE)

summary(fit9); coeftest(fit9)

We performed multiple dynamic regression models:

#Multiple regression analysis

cov1 <- c("precipta","deforest")

fit.mult1<- auto.arima(czs.ts[,"coefcases"], xreg = czs.ts[,cov1], stepwise=FALSE, approximation=FALSE)

summary(fit.mult1); coeftest(fit.mult1)

cov2 <- c("temp","forestcover")

fit.mult2<- auto.arima(czs.ts[,"coefcases"], xreg = czs.ts[,cov2], stepwise=FALSE, approximation=FALSE)

summary(fit.mult2); coeftest(fit.mult2)

cov3<- c("precipta","deforest","Ppov")

fit.mult3<- auto.arima(czs.ts[,"coefcases"], xreg = czs.ts[,cov3], stepwise=FALSE, approximation=FALSE)

summary(fit.mult3); coeftest(fit.mult3)

cov4<- c("precipta","deforest","MHDI")

fit.mult4<- auto.arima(czs.ts[,"coefcases"], xreg = czs.ts[,cov4], stepwise=FALSE, approximation=FALSE)

summary(fit.mult4); coeftest(fit.mult4)
