# Supplementary material for: Comparison of malaria incidence rates and socioeconomic-environmental factors between the states of Acre and Rondônia: a spatio-temporal modelling study
Source: Malar J. 2019 Sep 4;18:306. doi: 10.1186/s12936-019-2938-0 (PMC6727495; doi:10.1186/s12936-019-2938-0)
Supplement: Supplementary file 2 — Additional file 2. Interpolation of total precipitation and average maximum temperature. [file 12936_2019_2938_MOESM2_ESM.docx]

Interpolation of Total Precipitation and Average Maximum Temperature

Interpolations were done during the Scientific Initiation CNPq fellowship of GR, supervised by GZL, in 2017-2018, Centro Universitário Saúde ABC.

Briefly, we selected two meteorological parameters: 1) total precipitation (mm), 2) average maximum temperature (°C). Data for these parameters were available at meteorological stations throughout the Brazilian territory on the INMET website (http://www.inmet.gov.br/portal/). We used data from the following weather stations: Uruguaiana (-29.75, -57.08), Corumbá (-57.67, -19.02), Ponta Porã (-55.71, -22.55), Eirunepé (-69.86, -6.66), Labrea (-64.83, -7.25), Benjamin (-70.03, -4.38), CZS (-72.66, -7.6), RB (-70.76, -8.16) and Tarauacá (-67.8, -9.96). We summarized the total monthly precipitation and calculated the arithmetic mean for the average maximum temperature in the dry (May – Oct.) and rainy (Nov. – April) seasons of each year (2009-2015).

These data were imported into a geographic information system (QGIS v. 2.12.0) (http://www.qgis.org/en/site/). Subsequently, we performed inverse distance weighted interpolation method for building regular grids with 20-km^2^ as spatial resolution (200-m and 100-m pixel size). For each pixel we had an interpolated value of both climatic parameters. Zonal Statistics Plugin was utilized to calculate the mean value of precipitation and temperature per pixel in each municipality. As an example we show one of the outcomes (Figure 1).


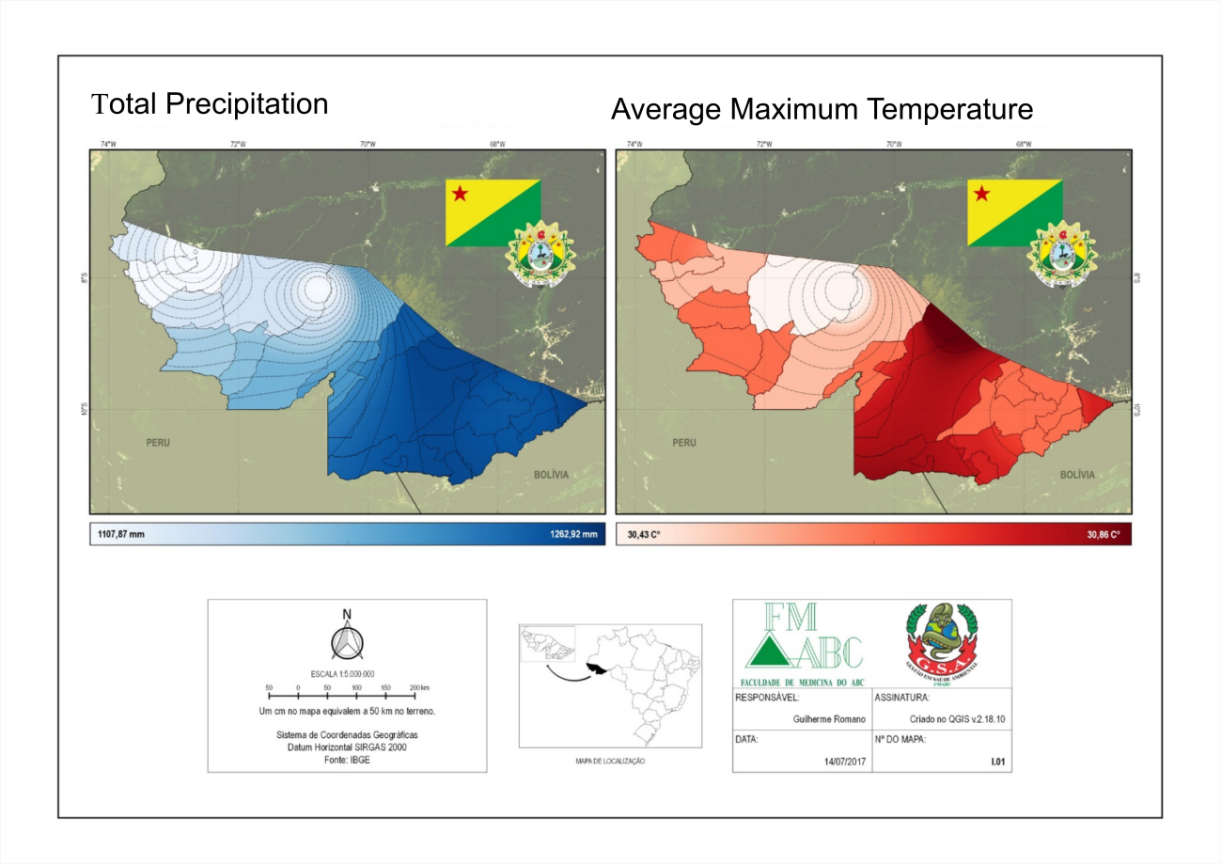


Figure 1 – Map produced by GR during the Scientific Initiation Program.
