# Supplementary material for: Comparison of malaria incidence rates and socioeconomic-environmental factors between the states of Acre and Rondônia: a spatio-temporal modelling study
Source: Malar J. 2019 Sep 4;18:306. doi: 10.1186/s12936-019-2938-0 (PMC6727495; doi:10.1186/s12936-019-2938-0)
Supplement: Supplementary file 3 — Additional file 3. Forest cover variations in municipalities of the states of Acre and Rondônia. [file 12936_2019_2938_MOESM3_ESM.docx]

Source: INPE/PRODES.
