# Supplementary material for: Comparison of malaria incidence rates and socioeconomic-environmental factors between the states of Acre and Rondônia: a spatio-temporal modelling study
Source: Malar J. 2019 Sep 4;18:306. doi: 10.1186/s12936-019-2938-0 (PMC6727495; doi:10.1186/s12936-019-2938-0)

**Principal Component Analysis full results**

**(1) Rondonia state – malaria incidence rate 2009 – 2015**

Table 1 - Importance of components

|  | PC1 | PC2 | PC3 | PC4 | PC5 | PC6 | PC7 |
| --- | --- | --- | --- | --- | --- | --- | --- |
| Standard deviation | 62.03 | 14.95 | 7.72 | 4.23 | 2.3 | 1.97 | 1.68 |
| Proportion of Variance | 0.93 | 0.05 | 0.01 | 0.004 | 0.001 | 0.001 | 0.001 |
| Cumulative Proportion | 0.93 | 0.98 | 0.99 | 0.997 | 0.998 | 0.999 | 1 |

Figure 1 – Plot and Biplot of PCA components


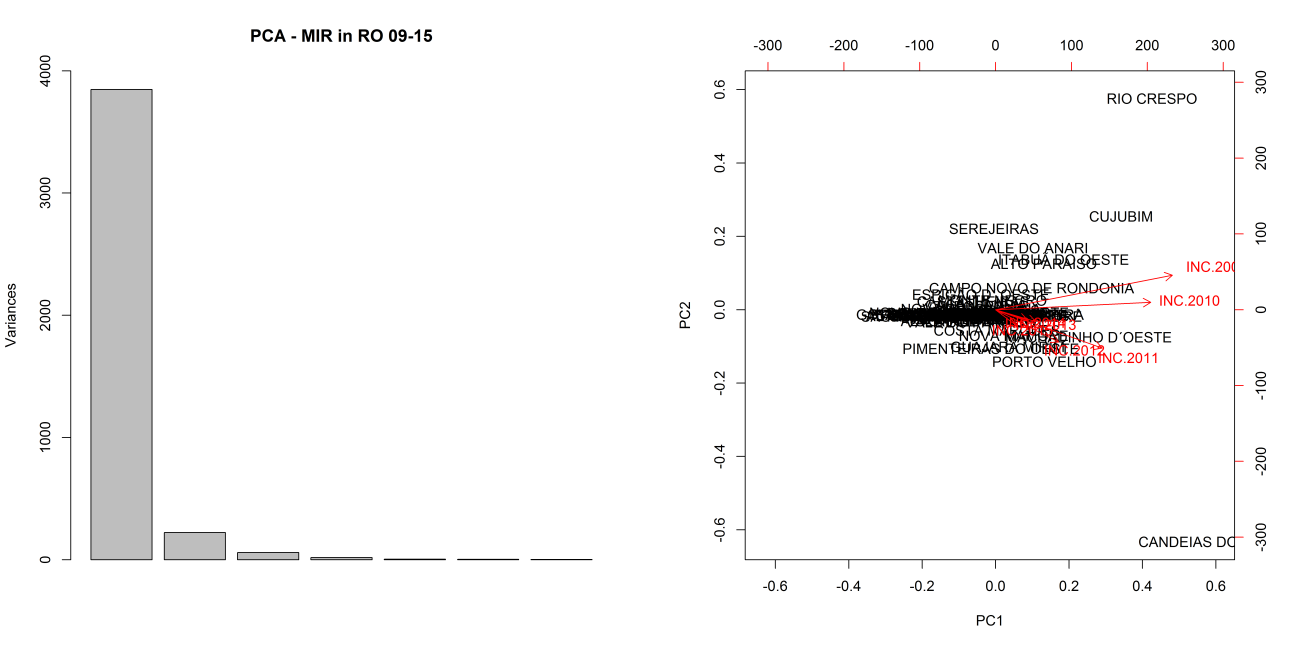


**(2) Rondonia state – annual deforestation rate 2009 – 2015**

Table 2 - Importance of components

|  | PC1 | PC2 | PC3 | PC4 | PC5 | PC6 | PC7 |
| --- | --- | --- | --- | --- | --- | --- | --- |
| Standard deviation | 3158.77 | 112.34 | 11.64 | 4.44 | 2.59 | 2.15 | 1.68 |
| Proportion of Variance | 0.9987 | 0.00126 | 0.00001 | 0.000 | 0.000 | 0.000 | 0.000 |
| Cumulative Proportion | 0.9987 | 0.99998 | 1 | 1 | 1 | 1 | 1 |

Figure 2 – Plot and Biplot of PCA components


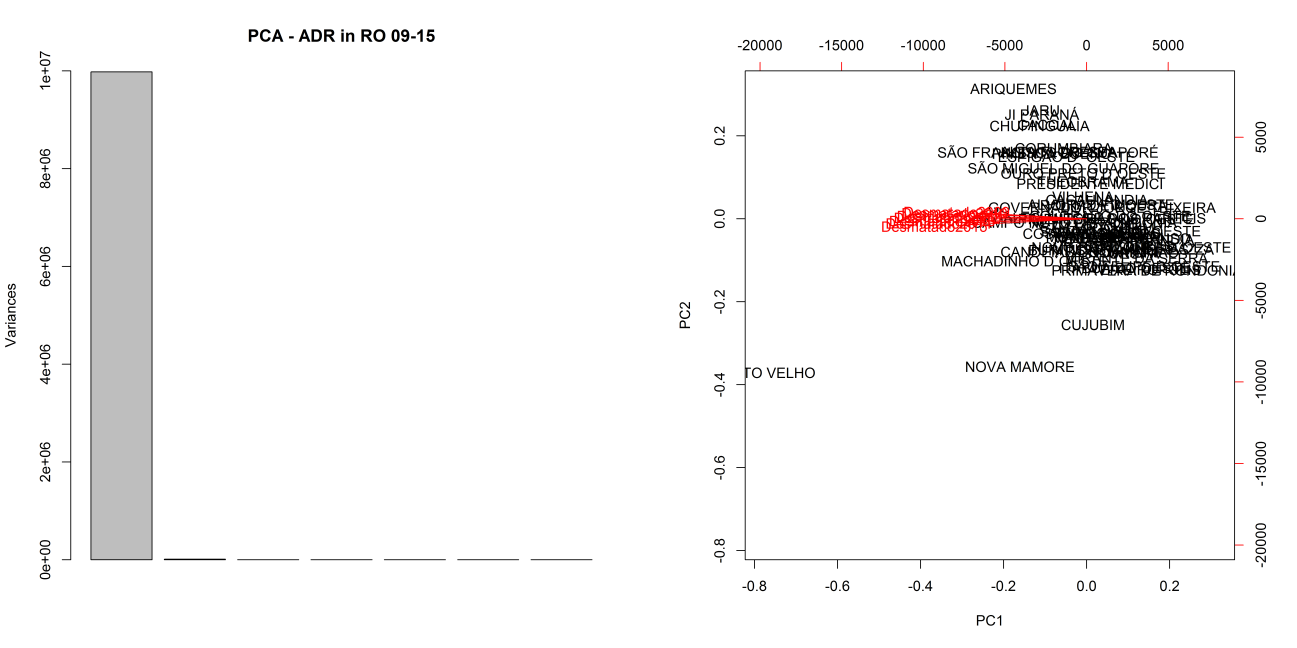


**(3) Acre state – malaria incidence rate 2009 – 2015**

Table 3 - Importance of components

|  | PC1 | PC2 | PC3 | PC4 | PC5 | PC6 | PC7 |
| --- | --- | --- | --- | --- | --- | --- | --- |
| Standard deviation | 258.11 | 22.17 | 14.66 | 7.61 | 5.87 | 3.92 | 2.98 |
| Proportion of Variance | 0.99 | 0.007 | 0.003 | 0.001 | 0.001 | 0.0002 | 0.0001 |
| Cumulative Proportion | 0.9878 | 0.995 | 0.998 | 0.999 | 0.9996 | 0.9998 | 1 |

Figure 3 – Plot and Biplot of PCA components


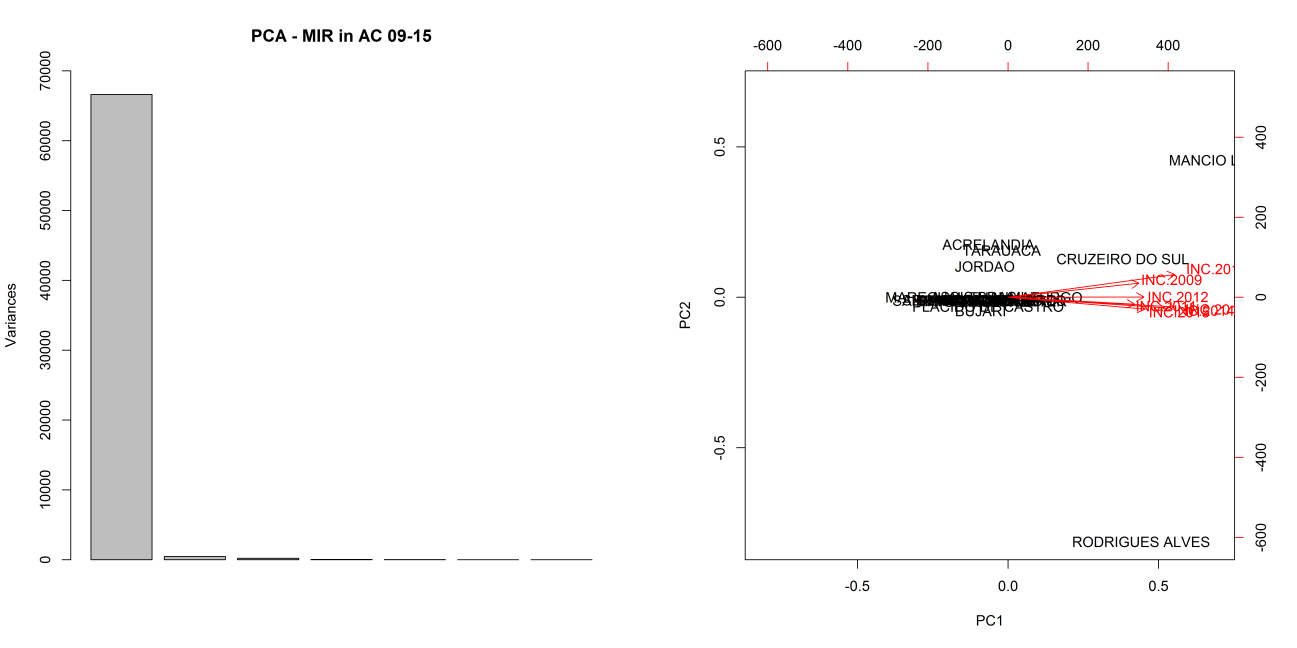


**(4) Acre state – annual deforestation rate rate 2009 – 2015**

Table 4 - Importance of components

|  | PC1 | PC2 | PC3 | PC4 | PC5 | PC6 | PC7 |
| --- | --- | --- | --- | --- | --- | --- | --- |
| Standard deviation | 1724.81 | 38.29 | 7.49 | 3.89 | 2.87 | 1.44 | 1.12 |
| Proportion of Variance | 0.9995 | 0.001 | 0.00002 | 0.00001 | 0 | 0 | 0 |
| Cumulative Proportion | 0.9995 | 0.99997 | 0.99999 | 1 | 1 | 1 | 1 |

Figure 4 – Plot and Biplot of PCA components


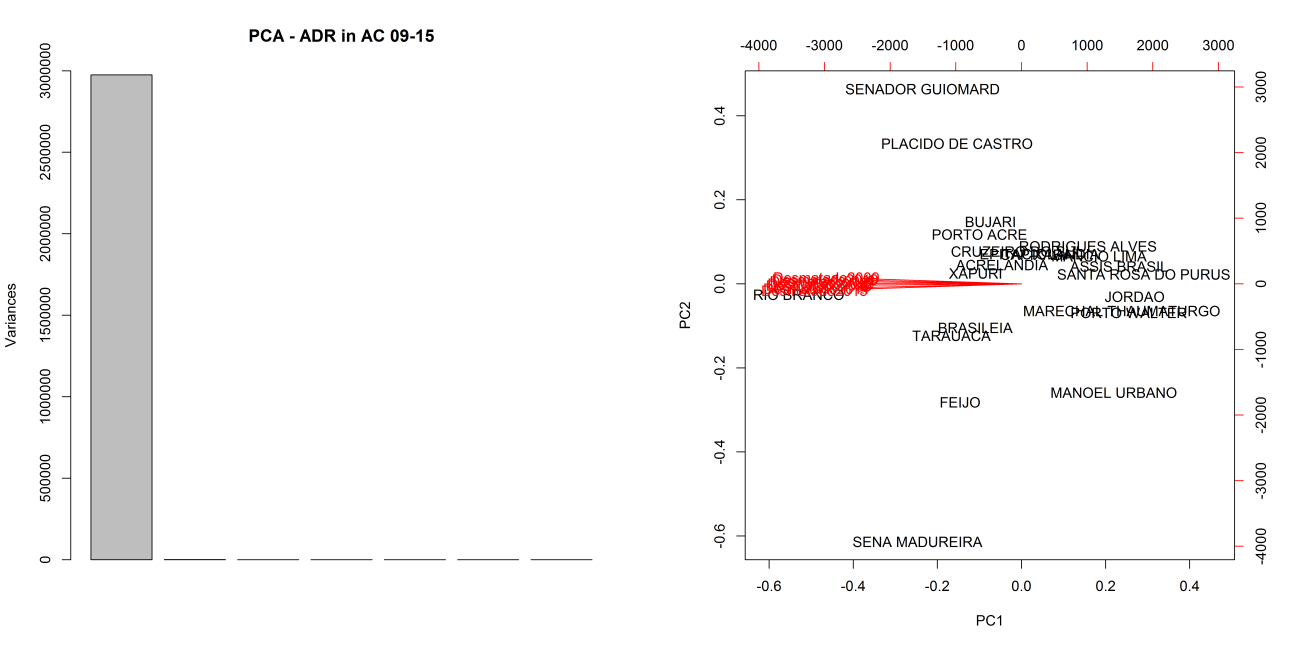

Supplement: Supplementary file 4 — Additional file 4. Results from the principal component analysis. [file 12936_2019_2938_MOESM4_ESM.docx]
