# Supplementary material for: Comparison of malaria incidence rates and socioeconomic-environmental factors between the states of Acre and Rondônia: a spatio-temporal modelling study
Source: Malar J. 2019 Sep 4;18:306. doi: 10.1186/s12936-019-2938-0 (PMC6727495; doi:10.1186/s12936-019-2938-0)
Supplement: Supplementary file 5 — Additional file 5. Results from the time-series modelling. [file 12936_2019_2938_MOESM5_ESM.docx]

Results from the time-series modeling

Cruzeiro do Sul municipality

Database 1A

Table 1A – Descriptive analysis

| **Variables** | **Mean (SD)** | **Min. – Max.** |
| --- | --- | --- |
| cases | 1432 (417) | 665 – 2610 |
| pop | 79247 (1524) | 76439 – 81475 |
| coefcases | 1808.9 (533) | 831 – 3337 |
| precipta | 1012 (417) | 439 – 1708 |
| temp | 31.72 (0.82) | 29.9 – 32.77 |
| forestcover | 7796 (271) | 7244 – 8142 |
| deforest | 999.8 (21.6) | 959 – 1025 |
| IMR | 21.69 (1.7) | 19.15 – 24.24 |
| ExtP | 16.35 (1.74) | 13.76 – 18.94 |
| Ppov | 31.55 (2.97) | 27.13 – 35.98 |
| gini | 0.64 (0.007) | 0.63 – 0.65 |
| MDHI | 0.69 (0.03) | 0.65 – 0.74 |

Figure 1A – Plotting with variables


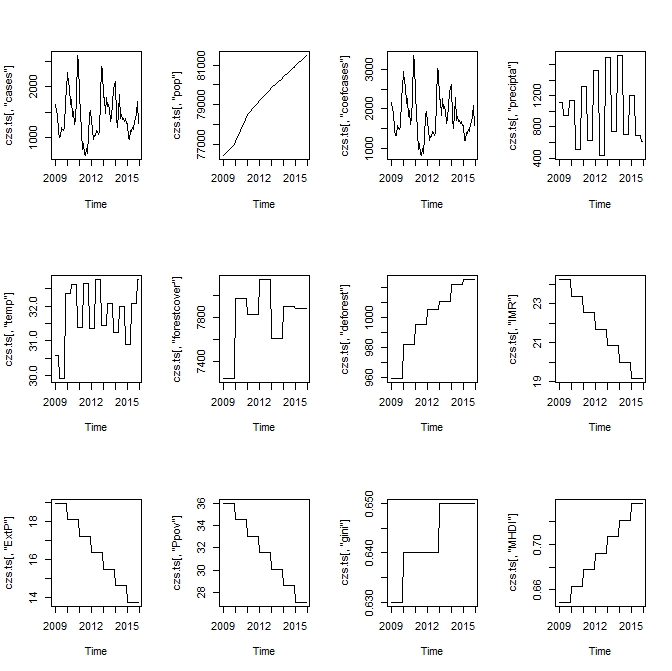


Table 2A – Augmented Dickey-Fuller Test

| **Response variable** | **Dickey-Fuller** | **Lag order** | **p-value** | **alternative hypothesis** |
| --- | --- | --- | --- | --- |
| coefcases | -3.4391 | 4 | 0.05455 | stationary |

Table 3A – Simple regression analysis

| **precipta** | Estimate | Std. Error | z value | Pr(>\|z\|) |
| --- | --- | --- | --- | --- |
| ar1 | 8.1407e-01 | 6.0353e-02 | 13.4885 | < 2.2e-16 |
| sar1 | 2.8030e-01 | 1.0649e-01 | 2.6321 | 0.008485 |
| sar2 | 2.3832e-01 | 1.3145e-01 | 1.8130 | 0.069834 |
| intercept | 1.5448e+03 | 3.1620e+02 | 4.8853 | 1.033e-06 |
| xreg | 2.3880e-01 | 1.6236e-01 | 1.4708 | 0.141339 |
|  |  |  |  |  |
| **temp** | Estimate | Std. Error | z value | Pr(>\|z\|) |
| ar1 | 0.820132 | 0.059828 | 13.7081 | < 2.2e-16 |
| sar1 | 0.312413 | 0.105076 | 2.9732 | 0.002947 |
| sar2 | 0.254722 | 0.128157 | 1.9876 | 0.046859 |
| xreg | 54.854424 | 9.851913 | 5.5679 | 2.578e-08 |
|  |  |  |  |  |
| **forestcover** | Estimate | Std. Error | z value | Pr(>\|z\|) |
| ar1 | 0.892387 | 0.054763 | 16.2955 | < 2.2e-16 |
| sma1 | -0.513075 | 0.124664 | -4.1157 | 3.861e-05 |
| xreg | 0.942366 | 0.244579 | 3.8530 | 0.0001167 |
|  |  |  |  |  |
| **deforest** | Estimate | Std. Error | z value | Pr(>\|z\|) |
| ar1 | 0.826353 | 0.058068 | 14.2309 | < 2.2e-16 |
| sar1 | 0.317779 | 0.105019 | 3.0259 | 0.002479 |
| sar2 | 0.249278 | 0.128377 | 1.9418 | 0.052165 |
| xreg | 1.768848 | 0.322927 | 5.4775 | 4.313e-08 |
|  |  |  |  |  |
| **IMR** | Estimate | Std. Error | z value | Pr(>\|z\|) |
| ar1 | 0.818169 | 0.059442 | 13.7641 | < 2.2e-16 |
| sar1 | 0.297415 | 0.105694 | 2.8139 | 0.004894 |
| sar2 | 0.258212 | 0.127419 | 2.0265 | 0.042715 |
| xreg | 81.360342 | 13.805941 | 5.8931 | 3.789e-09 |
|  |  |  |  |  |
| **ExtP** | Estimate | Std. Error | z value | Pr(>\|z\|) |
| ar1 | 0.820542 | 0.058966 | 13.9155 | < 2.2e-16 |
| sar1 | 0.295504 | 0.105893 | 2.7906 | 0.005261 |
| sar2 | 0.260335 | 0.127332 | 2.0445 | 0.040900 |
| xreg | 107.113463 | 18.435722 | 5.8101 | 6.243e-09 |
|  |  |  |  |  |

Table 3A – *Continuation*

| **Ppov** | Estimate | Std. Error | z value | Pr(>\|z\|) |
| --- | --- | --- | --- | --- |
| ar1 | 0.819331 | 0.059212 | 13.8373 | < 2.2e-16 |
| sar1 | 0.295812 | 0.105786 | 2.7963 | 0.005169 |
| sar2 | 0.259768 | 0.127344 | 2.0399 | 0.041361 |
| xreg | 55.712179 | 9.517326 | 5.8538 | 4.806e-09 |
|  |  |  |  |  |
| **gini** | Estimate | Std. Error | z value | Pr(>\|z\|) |
| ar1 | 8.2121e-01 | 5.8996e-02 | 13.9198 | < 2.2e-16 |
| sar1 | 3.1162e-01 | 1.0470e-01 | 2.9764 | 0.002917 |
| sar2 | 2.5444e-01 | 1.2754e-01 | 1.9951 | 0.046034 |
| xreg | 2.7562e+03 | 4.8660e+02 | 5.6642 | 1.477e-08 |
|  |  |  |  |  |
| **MHDI** | Estimate | Std. Error | z value | Pr(>\|z\|) |
| ar1 | 8.3074e-01 | 5.7427e-02 | 14.4660 | < 2.2e-16 |
| sar1 | 3.1833e-01 | 1.0506e-01 | 3.0301 | 0.002445 |
| sar2 | 2.5229e-01 | 1.2861e-01 | 1.9618 | 0.049791 |
| xreg | 2.5009e+03 | 4.8220e+02 | 5.1864 | 2.144e-07 |
|  |  |  |  |  |

Table 4A – Multiple regression analysis

| **Mult1** | Estimate | Std. Error | z value | Pr(>\|z\|) |
| --- | --- | --- | --- | --- |
| ar1 | 0.817995 | 0.059618 | 13.7207 | < 2.2e-16 |
| sar1 | 0.288401 | 0.106307 | 2.7129 | 0.006669 |
| sar2 | 0.235067 | 0.131694 | 1.7850 | 0.074269 |
| precipta | 0.237395 | 0.163241 | 1.4543 | 0.145874 |
| deforest | 1.548508 | 0.324102 | 4.7778 | 1.772e-06 |
|  |  |  |  |  |
| **Mult2** | Estimate | Std. Error | z value | Pr(>\|z\|) |
| ar1 | 8.5994e-01 | 5.2043e-02 | 16.5237 | < 2.2e-16 |
| sar1 | 5.3164e-01 | 8.9069e-02 | 5.9689 | 2.389e-09 |
| intercept | -4.3209e+03 | 2.6298e+03 | -1.6430 | 0.1004 |
| temp | -1.8923e+01 | 6.6768e+01 | -0.2834 | 0.7769 |
| forestcover | 8.6624e-01 | 2.1745e-01 | 3.9836 | 6.787e-05 |
|  |  |  |  |  |
| **Mult3** | Estimate | Std. Error | z value | Pr(>\|z\|) |
| ar1 | 8.3464e-01 | 6.0603e-02 | 13.7721 | < 2.2e-16 |
| sar1 | 4.2674e-01 | 1.0330e-01 | 4.1309 | 3.613e-05 |
| intercept | -5.1514e+04 | 2.3408e+04 | -2.2006 | 0.02776 |
| precipta | 2.3618e-01 | 1.5819e-01 | 1.4931 | 0.13542 |
| deforest | 4.2239e+01 | 1.8909e+01 | 2.2339 | 0.02549 |
| Ppov | 3.4620e+02 | 1.5637e+02 | 2.2140 | 0.02683 |
|  |  |  |  |  |

Table 4A – *Continuation*

| **Mult4** | Estimate | Std. Error | z value | Pr(>\|z\|) |
| --- | --- | --- | --- | --- |
| ar1 | 8.3724e-01 | 6.0355e-02 | 13.8718 | < 2.2e-16 |
| sar1 | 4.3449e-01 | 1.0276e-01 | 4.2283 | 2.355e-05 |
| intercept | -1.6070e+04 | 9.6072e+03 | -1.6727 | 0.09438 |
| precipta | 2.3354e-01 | 1.5860e-01 | 1.4725 | 0.14089 |
| deforest | 4.2729e+01 | 1.7823e+01 | 2.3975 | 0.01651 |
| MHDI | -3.6113e+04 | 1.5154e+04 | -2.3830 | 0.01717 |
|  |  |  |  |  |

Mâncio Lima municipality

Database 1B

Table 1B – Descriptive analysis

| **Variables** | **Mean (SD)** | **Min. – Max.** |
| --- | --- | --- |
| cases | 477 (186) | 217 – 990 |
| pop | 15778 (809) | 14417 – 17144 |
| coefcases | 3023 (1176) | 1440 – 6509 |
| precipta | 1017 (417) | 451 – 1707 |
| temp | 31.67 (0.82) | 30 – 32.77 |
| forestcover | 5115 (106) | 4948 – 5283 |
| deforest | 407.2 (9.96) | 390 – 418 |

Figure 1B – Plotting with variables


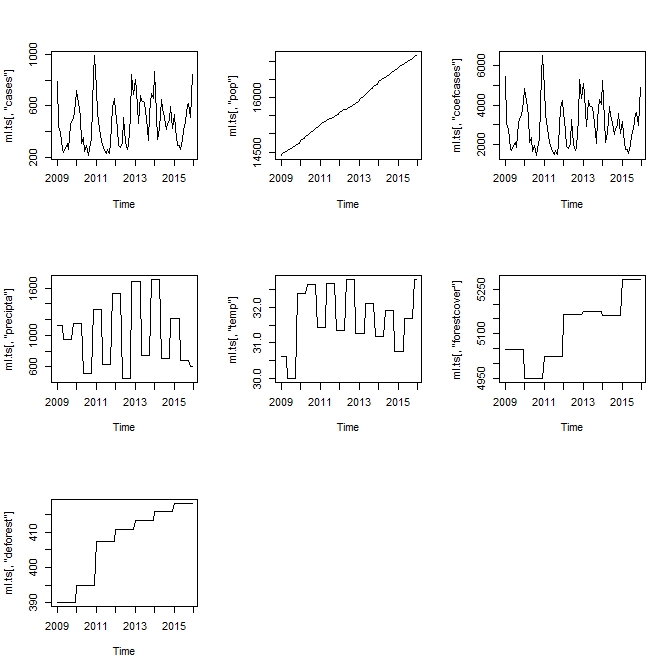


Table 2B – Augmented Dickey-Fuller Test

| **Response variable** | **Dickey-Fuller** | **Lag order** | **p-value** | **alternative hypothesis** |
| --- | --- | --- | --- | --- |
| coefcases | -3.8747 | 4 | 0.0194 | stationary |

Table 3B – Multiple regression analysis

| **Mult1** | Estimate | Std. Error | z value | Pr(>\|z\|) |
| --- | --- | --- | --- | --- |
| ar1 | 0.620113 | 0.092895 | 6.6754 | 2.465e-11 |
| sma1 | 0.420164 | 0.102708 | 4.0908 | 4.298e-05 |
| precipta | 0.875070 | 0.417156 | 2.0977 | 0.03593 |
| deforest | 5.439960 | 1.323273 | 4.1110 | 3.940e-05 |
|  |  |  |  |  |

Rodrigues Alves municipality

Database 1C

Table 1C – Descriptive analysis

| **Variables** | **Mean (SD)** | **Min. – Max.** |
| --- | --- | --- |
| cases | 313.6 (110) | 121 – 641 |
| pop | 15047 (1171) | 13054 – 16936 |
| coefcases | 2074 (694) | 856 – 4493 |
| precipta | 1012.2 (415) | 436.4 – 1704.3 |
| temp | 31.70 (0.83) | 29.84 – 32.77 |
| forestcover | 2570 (163) | 2270 – 2706 |
| deforest | 487.4 (9.99) | 467.9 – 498.3 |

Figure 1C – Plotting with variables


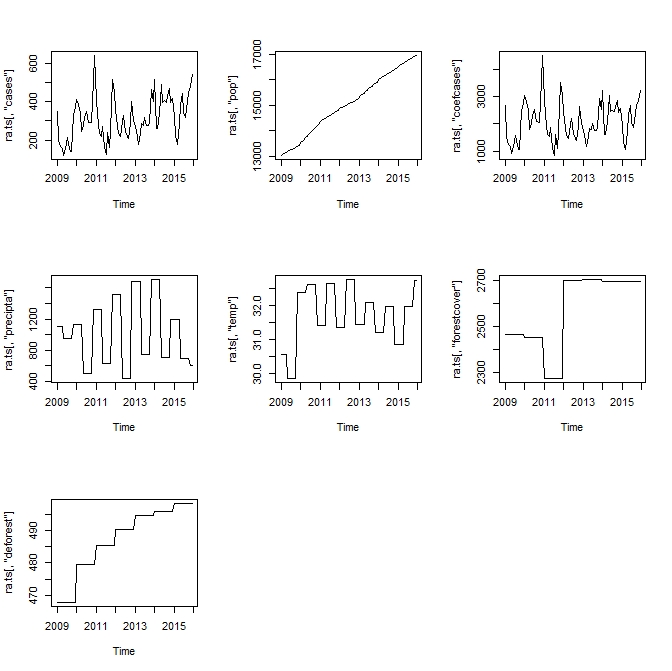


Table 2C – Augmented Dickey-Fuller Test

| **Response variable** | **Dickey-Fuller** | **Lag order** | **p-value** | **alternative hypothesis** |
| --- | --- | --- | --- | --- |
| coefcases | -3.4107 | 4 | 0.05917 | stationary |

Table 3C – Multiple regression analysis

| **Mult1** | Estimate | Std. Error | z value | Pr(>\|z\|) |
| --- | --- | --- | --- | --- |
| ar1 | 0.6151134 | 0.0877869 | 7.0069 | 2.437e-12 |
| sar1 | 0.3118390 | 0.1101982 | 2.8298 | 0.004658 |
| sar2 | 0.2736658 | 0.1285951 | 2.1281 | 0.033327 |
| precipta | 0.0085515 | 0.2846758 | 0.0300 | 0.976036 |
| deforest | 4.3277895 | 0.7640205 | 5.6645 | 1.475e-08 |
|  |  |  |  |  |

Tarauaca municipality

Database 1D

Table 1D – Descriptive analysis

| **Variables** | **Mean (SD)** | **Min. – Max.** |
| --- | --- | --- |
| cases | 100.5 (70.8) | 5 – 340 |
| pop | 36318 (1632) | 33408 – 38771 |
| coefcases | 280 (204) | 14 – 1018 |
| precipta | 1027 (502) | 443 – 1831 |
| temp | 31.81 (0.75) | 30.43 – 32.90 |
| forestcover | 19251 (364) | 18694 – 19703 |
| deforest | 1480 (44.7) | 1410 – 1537 |

Figure 1D – Plotting with variables


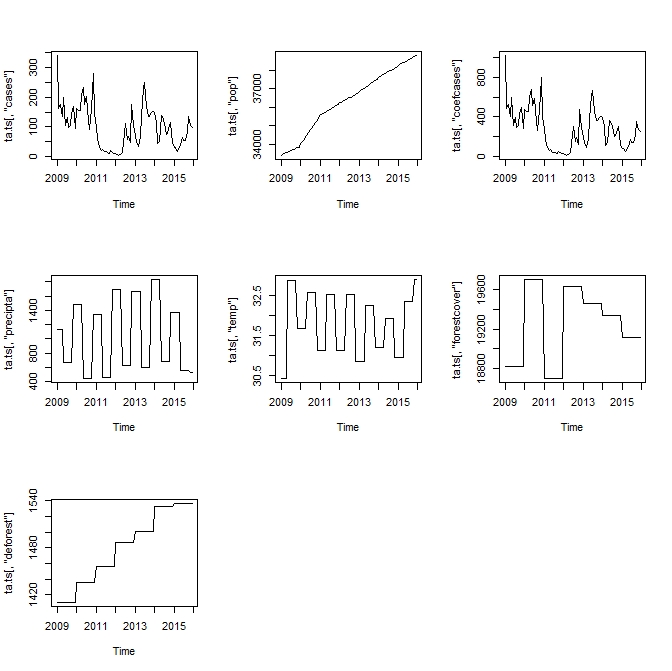


Table 2D – Augmented Dickey-Fuller Test

| **Response variable** | **Dickey-Fuller** | **Lag order** | **p-value** | **alternative hypothesis** |
| --- | --- | --- | --- | --- |
| coefcases | -2.3989 | 4 | 0.4123 | stationary |

Table 3D – Multiple regression analysis

| **Mult1** | Estimate | Std. Error | z value | Pr(>\|z\|) |
| --- | --- | --- | --- | --- |
| ar1 | 0.870553 | 0.061008 | 14.2695 | < 2e-16 |
| sar1 | 0.218720 | 0.109147 | 2.0039 | 0.04508 |
| sar2 | 0.318671 | 0.137299 | 2.3210 | 0.02029 |
| precipta | 0.012348 | 0.060446 | 0.2043 | 0.83814 |
| deforest | 0.232967 | 0.123553 | 1.8856 | 0.05935 |
|  |  |  |  |  |

Porto Walter municipality

Database 1E

Table 1E – Descriptive analysis

| **Variables** | **Mean (SD)** | **Min. – Max.** |
| --- | --- | --- |
| cases | 36.2 (32.5) | 2 – 136 |
| pop | 9635 (644) | 8585 – 10735 |
| coefcases | 253 (140) | 34 – 757 |
| precipta | 1023 (431) | 488 – 1731 |
| temp | 31.7 (0.74) | 30.57 – 32.79 |
| forestcover | 6119 (407) | 5228 – 6478 |
| deforest | 191 (14.72) | 164 – 208 |

Figure 1E – Plotting with variables


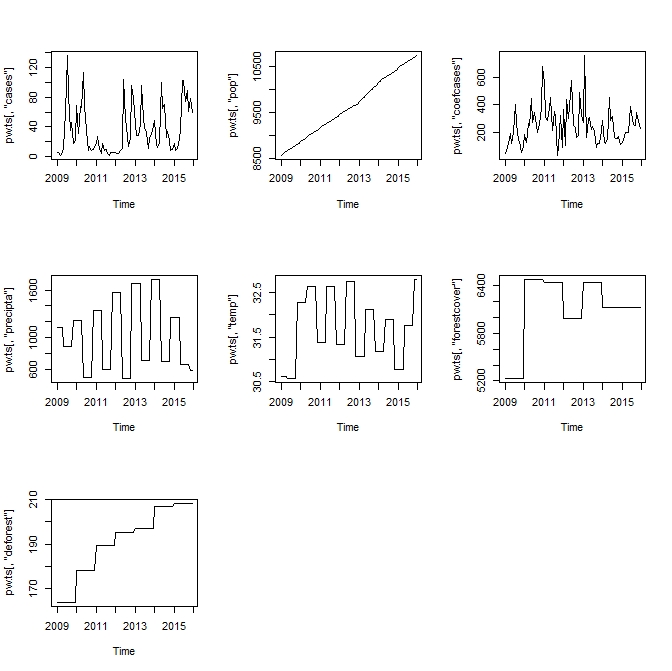


Table 2E – Augmented Dickey-Fuller Test

| **Response variable** | **Dickey-Fuller** | **Lag order** | **p-value** | **alternative hypothesis** |
| --- | --- | --- | --- | --- |
| coefcases | -3.4917 | 4 | 0.04784 | stationary |

Table 3E – Multiple regression analysis

| **Mult1** | Estimate | Std. Error | z value | Pr(>\|z\|) |
| --- | --- | --- | --- | --- |
| ar1 | 0.3351130 | 0.1030148 | 3.2531 | 0.001142 |
| precipta | -0.0017576 | 0.0411781 | -0.0427 | 0.965955 |
| deforest | 1.3238317 | 0.2453887 | 5.3948 | 6.859e-08 |
|  |  |  |  |  |
